# Endoscopic Retrieval of a Migrated Plastic Pigtail Stent in a Patient With Gastric Bypass Surgery Using Novel Endoscopic Cutting Device

**DOI:** 10.14309/crj.0000000000002185

**Published:** 2026-06-11

**Authors:** Mohammad Shahzaib Qadir, Arsalan Syed, Ridhima Kaul, Arjun Chatterjee, Jerry Dang, Hassan Siddiki

**Affiliations:** 1 Department of Gastroenterology, Hepatology and Nutrition, Cleveland Clinic Foundation, Cleveland, OH; 2 Department of Internal Medicine, Cleveland Clinic Foundation, Cleveland, OH; 3 Department of Surgery, Cleveland Clinic Foundation, Cleveland, OH

## CASE REPORT

**Figure 1.**
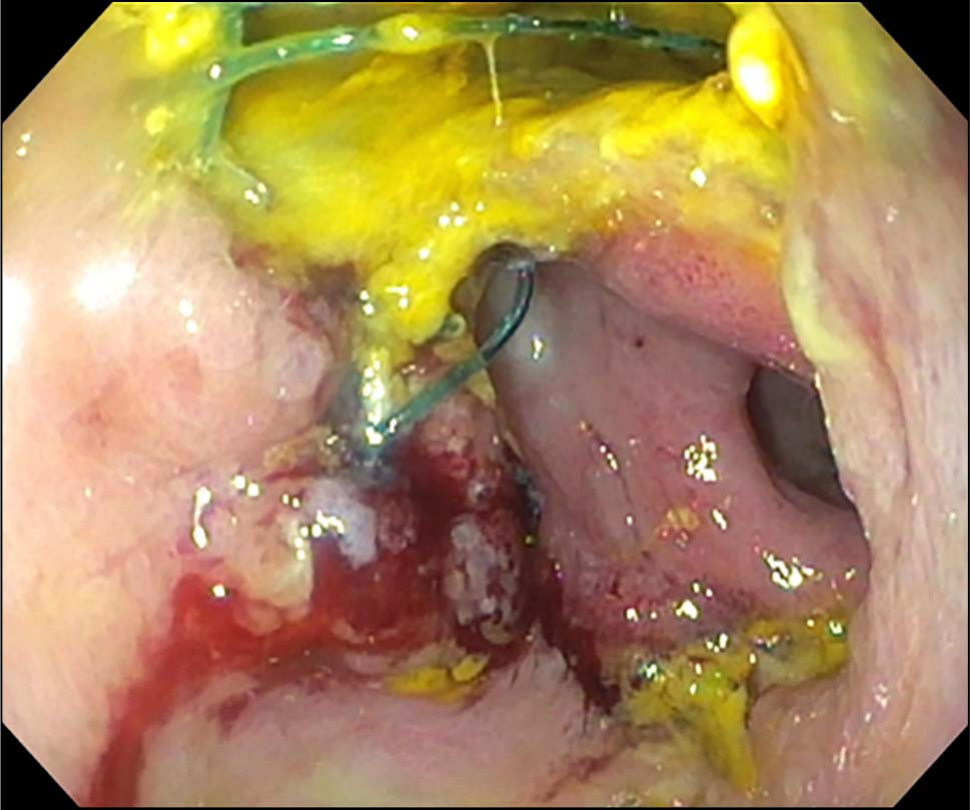
Endoscopy showing dense scar tissue at the gastrojejunal anastomosis without visible stent.

**Figure 2.**
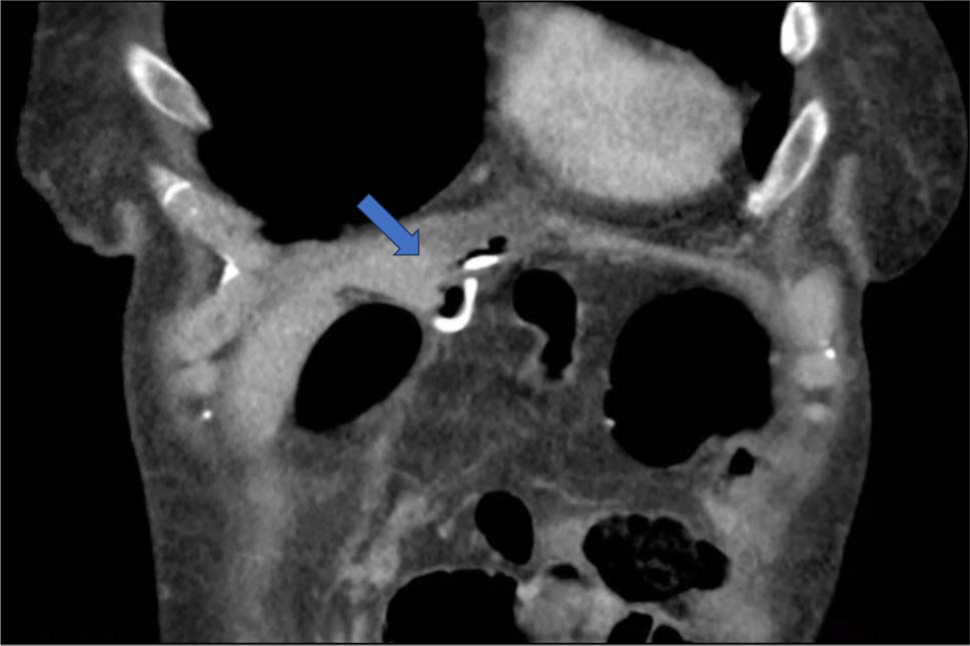
Computed tomography of the abdomen revealed migration of the stent into a perigastric sinus tract.

**Figure 3.**
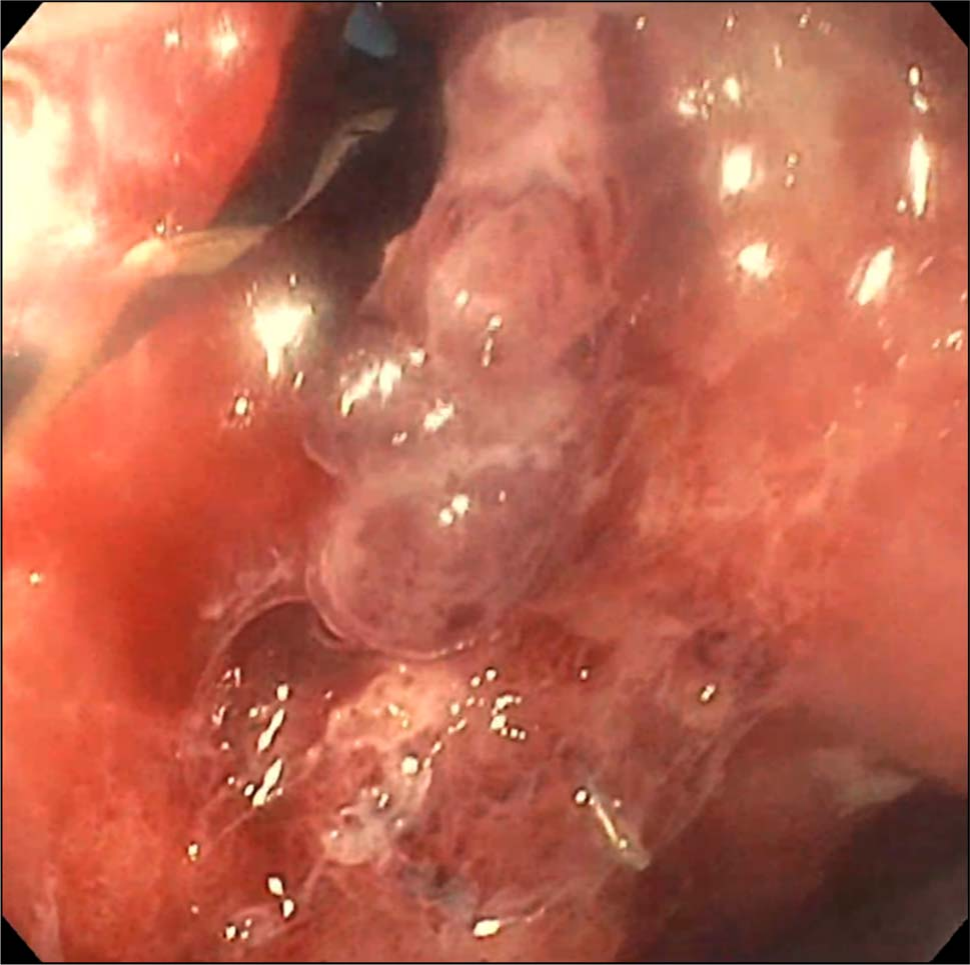
Failed retrieval attempts using catheter-based access, balloon dilation, and grasping devices before use of bipolar scissors.

Gastrojejunal anastomotic leaks are a recognized complication of Roux-en-Y gastric bypass and are often managed with transmural plastic stent placement; however, stent migration can occur and retrieval is challenging in altered anatomy.^1–4^ We present a 52-year-old woman with previous Roux-en-Y gastric bypass who developed a gastrojejunal staple-line leak complicated by a perigastric collection, managed with a 7-Fr double-pigtail stent. During planned removal, the stent was not visualized endoscopically (Figure 1); the staple line demonstrated dense scar tissue without clear disruption. Computed tomography revealed migration into a perigastric sinus tract with interval healing of the anastomosis (Figure 2). Endoscopic ultrasound and fluoroscopy localized the tract, and a guidewire was advanced. Multiple conventional retrieval techniques, including catheter-based access (sphincterotome), balloon dilation, biopsy forceps, and grasping devices, were unsuccessful (Figure 3). Bipolar radiofrequency scissors were then used for controlled dissection of the sinus tract, allowing direct visualization and successful removal of the stent. Final contrast injection confirmed closure without leak (Video 1). The patient had no immediate or delayed adverse events and remained asymptomatic on follow-up. This case highlights the importance of EUS-fluoroscopic guidance in altered anatomy and demonstrates that bipolar scissors provide a precise and effective option for retrieval of embedded prostheses when standard techniques fail.^5^

## DISCLOSURES

Author contributions: MS Qadir, A. Syed, R. Kaul, and A. Chatterjee: Drafting the article; A. Chatterjee: Video editing and narration; J. Dang, and H. Siddiki: Final approval of manuscript and is the article guarantor.

Financial disclosure: None to report.

Informed consent was obtained for this case report.

## References

[1] Gonzalez R Sarr MG Smith CD Diagnosis and contemporary management of anastomotic leaks after gastric bypass for obesity. J Am Coll Surg. 2007;204(1):47–55. 17189112 10.1016/j.jamcollsurg.2006.09.023

[2] Chatterjee A Singh A Gurajala R Chahal P. Iatrogenic portobiliary fistula. Am J Gastroenterol. 2023;118(4).597. 36729791 10.14309/ajg.0000000000002092

[3] Chatterjee A Singh A Garg R Chahal P. Hepatic perforation from migrated biliary stents. Gastrointest Endosc. 2023;98(5):865–6. 37406890 10.1016/j.gie.2023.06.037

[4] Cymbal M Chatterjee A Prado R Unraveling the complications: Stent migration and duodenal fistula in a metastatic desmoplastic round cell tumor. ACG Case Rep J. 2026;13(2):e01997. 41647150 10.14309/crj.0000000000001997 PMC12871935

[5] Kaul R Chatterjee A Prado R Siddiki H. An endoscopic approach to a pouch stricture after failed needle knife stricturotomy. ACG Case Rep J. 2026;13(6):e02161. 42238719 10.14309/crj.0000000000002161 PMC13229455

